# Effectiveness of the Specific Mobility Exercises on Pain Intensity and Quality of Life Among Stoma Patients: A Quasi-experimental Study

**DOI:** 10.7759/cureus.63715

**Published:** 2024-07-02

**Authors:** Thoufeeq Fathima Abdul Khadar, Vinodhkumar Ramalingam

**Affiliations:** 1 Physiotherapy, Saveetha College of Physiotherapy, Saveetha Institute of Medical and Technical Sciences, Saveetha University, Chennai, IND

**Keywords:** physical therapy, pain, quality of life (qol), colorectal cancer, inflammatory bowel disease

## Abstract

Background: An intestinal stoma is a surgically created artificial opening in the abdominal wall that helps the large or small intestine end to divert the faecal matter for stoma patients with an underlying condition of inflammatory bowel disease and colorectal cancer. When a stoma is formed following surgery, one of the difficulties stoma patients confront has been identified as prolonged immobilization, which can eventually result in muscle inactivity that results due to their illness. Patients with stoma often experience an increase in pain and a decrease in quality of life. Patients can be mobilized and their muscles can be activated with the help of an early intervention called specific mobility exercises.

Aim: The present study aimed to explore the specific mobility exercises that reduce pain and improve quality of life among stoma patients.

Methodology: This quasi-experimental study involved 21 patients who underwent stoma surgery and were selected according to the inclusion and exclusion criteria. The experimental procedures were explained to all the patients and their written informed consent was obtained. The patients performed specific mobility exercises for 30 minutes per day. Treatment was given for four weeks every day after three to four days of stoma surgery. The patient's pain and quality of life were assessed using the Numerical Pain Rating Scale and the Stoma-Quality of Life (QoL) Questionnaire and pre-test and post-test values were recorded before and after the exercises. The data were tabulated and evaluated.

Results: The findings suggest that specific mobility exercises following four weeks of intervention have a significant effect (p< 0.001) in reducing pain except in young adult stoma patients as they were found to be anxious and depressed, which was reflected in the findings as not statistically significant for pain on the NPRS (t(1) = 7, p > 0.001). However, it has been demonstrated that these specific mobility exercises have a significant effect (p< 0.001) in improving the quality of life among all stoma patients.

Conclusion: The study evidenced that four weeks of specific mobility exercises in line with general medical treatment showed a significant reduction in pain and an improvement in quality of life among stoma patients. However, it should be noted that in the study, the majority of stoma patients were male and there were only a few patients with inflammatory bowel disease, which can limit the study findings. Future studies have to focus on equally distributing gender and conditions by emphasizing the importance of randomizing patients into the experimental and control groups and involving a combination of other exercises in rehabilitation for patients following stoma surgery.

## Introduction

An intestinal stoma is a small opening created surgically in the abdominal wall to bring out the end of either the large intestine known as colostomy or the small intestine known as an ileostomy, allowing for the diversion of waste products [1]. The most common underlying conditions that require ostomy surgery are Crohn’s disease, ulcerative colitis, and colitis, which are all inflammatory bowel diseases and the other condition that needs ostomy surgery is colorectal cancer [2]. Inflammatory bowel disease is a group of autoimmune diseases that develop as a result of an inappropriate immune response to gut microflora and food. It is considered to be one of the chronic inflammatory diseases and its cause is not known. Although it can appear at any age, it is typically diagnosed between the ages of 20 and 40 years. Any part of the gastrointestinal tract can be affected by Crohn’s disease. Ileitis is common in women, but ulcerative colitis affects both genders equally. However, this condition increases the risk of colorectal cancer eventually [3]. A major public health concern is colorectal cancer. Colorectal cancer ranks globally as the fourth most prevalent cancer causing death and the third most common cancer diagnosis [4]. The first-line treatments that are frequently used to treat bowel cancer are adjuvant chemotherapy and radiation therapy, while anti-inflammatory medications are frequently used for treating inflammatory bowel disease. Bowel cancer typically strikes older adults in contrast to inflammatory bowel disease, which typically affects young adults [5]. Within a month following surgery, early complications of the stoma include peristomal irritation, stoma infection, ischemia, high output stoma, and retraction, while the parastomal hernia, prolapse, and stenosis are considered to be late complications, which are commonly seen among ostomy patients [6].

A parastomal hernia is a formation of a bulge around the stoma caused by the protrusion of a loop of one or more bowels through the anterior abdominal wall. It is caused by weakness in the abdominal wall following stoma surgery, which leads to the creation of another weak area in the abdominal wall. Most cases of parastomal hernia happen to appear within two years following stoma surgery [7]. The risk factors for the parastomal hernia are age, especially those who are aged 60 years and older (predominantly females), waist circumference greater than 100 cm, ostomy type, diabetes mellitus, and chronic obstructive pulmonary disease [8]. For the prevention of parastomal hernia, patients were provided with supportive garments, thorough guidance on lifting, and intervention of abdominal exercises, which can be performed easily, which consequently improved their quality of life [9].

In the human body, deep abdominal muscles create a muscular band that tightens like a corset, which helps to provide support to the spine and the abdominal area. After undergoing abdominal surgery, abdominal physiology gets altered and causes atrophy in the midline muscular wall and damage to the nerve supply, reducing physical activity [10]. It is a known fact that following surgery, the abdominal muscles become weak, and this is an important problem to be addressed by the physiotherapist by choosing the right exercise. Exercise is vital for both physical and mental well-being, as it prevents the worsening of the patient's condition and helps them in regaining confidence. An intervention that involves appropriate physical activity for rehabilitation for physical recovery is important after an illness or surgery. Providing exercise is secure during and after chemotherapy for cancer and leads to enhancements in physical functioning, fatigue, and quality of life [11,12]. So, individuals who have ostomies need specific care during the recovery and rehabilitation phases to improve their physical activity and maintain their physical well-being and quality of life [13].

One of the difficulties stoma patients confront has been identified as prolonged immobilization, which can eventually result in muscle inactivity that results due to their illness. Patients with stomas often experience an increase in pain and a decrease in quality of life. Patients can be mobilized and their muscles can be activated with the use of an early intervention called specific mobility exercises. The main goal of these exercises is to keep patients physically active. Walking has been shown to improve health-related quality of life, along with which abdominal muscle activation exercises can be given. The intensity of exercises must be gradually increased taking the patient’s post-operative condition into consideration [14-16]. Also, as studies related to the rehabilitation following stoma surgery are very few, we aim to find the effectiveness of specific mobility exercises in reducing pain and improving the quality of life among stoma patients. 

## Materials and methods

Subjects

This quasi-experimental study involved 21 patients who underwent stoma surgery. Patients were identified by a convenience sampling method in Saveetha Medical College and Hospital (SMCH). They were included in the study based on the inclusion and exclusion criteria. During the study, the Numerical Pain Rating Scale and Stoma-Quality of Life (QoL) Questionnaire were used for assessment. Experimental procedures were explained to all the patients before obtaining their informed consent.

Inclusion criteria: (i) 18 years and above; (ii) both male and female; (iii) patients who underwent stoma surgery after being diagnosed with inflammatory bowel disease and colorectal cancer.

Exclusion criteria: (i) pregnant and breast-feeding women; (ii) cognitively incapable of giving informed consent; (iii) exercises prohibited due to any complication or restriction; (iv) intensive care unit (ICU) stay and performing any exercises prohibited by medical condition; (v) emergency surgery for stoma formation.

Procedure

A total of 21 patients were selected for the study and they performed specific mobility exercises. The exercises were undertaken by the patients for 30 minutes every day for four weeks. They progressed with their exercises to their maximum ability for a better quality of life. Specific mobility exercises were given to them two times per day with 10 repetitions after three to four days of their surgery, along with walking for five to eight minutes. They were given exercises only if the resting pain score on the Numerical Pain Rating Scale was below 3 in the early postoperative period.

During the first two weeks, they were introduced to diaphragmatic breathing, ankle toe movements, ankle circles, heel slides, pelvic rotation, abdominal drawing-in maneuver, pelvic bridging, knee lift in crook lying, straight leg raising, knee and ankle isometrics, and diagonal isometric press. In the sitting posture, they were introduced to abdominal drawing-in maneuver, arm raising, knee lifting, sit-to-stand activities, and walking for five to eight minutes.

In the third and fourth weeks, they were introduced to diaphragmatic breathing, diagonal isometric press, abdominal drawing-in maneuver in quadruped, bird dog exercise, ball squeeze, knee lift and arm raise on ball, abdominal drawing-in maneuver on ball and in knee plank, arm swing, step up, and walking for five to eight minutes.

When they were not able to do straight-leg raise by themselves, it was modified with a towel. While performing these specific mobility exercises such as bridging, quadruped position, and walking, the patients were assisted.

Outcome measures

Stoma-QoL Questionnaire

The widely used, valid, and reliable Stoma-QoL Questionnaire was created to assess patients’ quality of life after stoma surgery. It consists of 20 questions covering the following topics: intimate relationships, sleeping issues, and relationships with family, close friends, and non-family members [17]. The scale has four points: (1) Always; (2) Sometimes; (3) Rarely; (4) Not at all. Answers are required for each question, and the total possible score is between 20 and 80. A higher score denotes a greater quality of life (Table 1).

**Table 1:** Stoma-QoL Questionnaire

S.no	Stoma-QoL Questionnaire	Always	Sometimes	Rarely	Not at all
1	I become anxious when the pouch is full				
2	I worry that the pouch will loosen				
3	I feel the need to know where the nearest toilet is				
4	I worry that the pouch may smell				
5	I worry about noises from the stoma				
6	I need to rest during the day				
7	My stoma pouch limits the choice of clothes that I can wear				
8	I feel tired during the day				
9	My stoma makes me feel sexually unattractive				
10	I sleep badly during the night				
11	I worry that the pouch rustles				
12	I feel embarrassed about my body because of my stoma				
13	It would be difficult for me to stay away from home overnight				
14	It is difficult to hide the fact that I wear a pouch				
15	I worry that my condition is a burden to people close to me				
16	I avoid close physical contact with my friends				
17	My stoma makes it difficult for me to be with other people				
18	I am afraid of meeting new people				
19	I feel lonely even when I am with other people				
20	I worry that my family feels awkward around me				

Numerical Pain Rating Scale

The most basic and widely used scale is the Numerical Pain Rating Scale (NPRS). Scores range from 0 to 10 on this most popular scale, with no pain denoted by a score of zero and the worst pain imaginable represented by 10. Most of the time, the person experiencing pain selects a number that best captures the severity of their pain by drawing a circle around it [18].

Statistical analysis

Using descriptive and inferential analysis, the collected data were tabulated and evaluated. The mean and standard deviation (SD) were calculated for all the parameters. The statistically significant differences between pre-test and post-test measures were examined using a paired t-test for Numerical Pain Rating Scale and Stoma-QoL Questionnaire. A p value less than 0. 05 was considered statistically significant. The software program used for the data analysis was IBM SPSS Statistics, version 27 (IBM Corp., Armonk, NY).

## Results

A total of 21 patients were included in the study, out of which 16 were male and five were female. Seven patients underwent surgery after being diagnosed with inflammatory bowel disease and 14 patients underwent surgery after they were diagnosed with colorectal cancer. Only patients who underwent elective procedures were selected. We classified the patients according to their age category. Young adults belong to the age group of 18-25 years, adults were aged 26-44 years, middle-aged adults were aged 45-59 years, and older adults were aged 60 years and above.

There were two males in young adult patients. In adults, there were a total of five patients, out of which two were males and three were females. In middle-aged adults, there were 10 males and one female in a total of 11 patients. In older adults, there were two males and one female in a total of three patients (Table 2).

**Table 2:** Descriptive data of the participants in terms of gender

Age category	Gender	Frequency	%
Young adults (18-25 years old)	Male	2	100
Adults (26-44 years old)	Male	2	40
	Female	3	60
Middle-aged adults (45-59 years old)	Male	10	90
	Female	1	91
Older adults (60 and above)	Male	2	66.7
	Female	1	33.3

Patients were classified according to the condition in which they underwent stoma surgery after being diagnosed with inflammatory bowel disease. Out of seven such patients, there were two young adults and five adults (Table 3).

**Table 3:** Number of patients who underwent surgery after being diagnosed with inflammatory bowel disease

Inflammatory bowel disease	Frequency	%
Young adults (18-25 years old)	2	100
Adults (26-44 years old)	5	100

Fourteen patients underwent stoma surgery after being diagnosed with colorectal cancer, of which 11 were middle-aged adults and three were older adults (Table 4).

**Table 4:** Number of patients who underwent surgery after being diagnosed with colorectal cancer

Colorectal cancer	Frequency	%
Middle-aged adults (45-59 years)	11	100
Older adults (60 yeaers and above)	3	100

The pre-test (7.5±0.7) and post-test (4.0±0.0) values of pain intensity among young adult stoma patients were not statistically significant (t(1) = 7, p > 0.001). But the pain intensity of adult stoma patients showed a significant reduction from pre-test (6.2±0.83) to post-test (3.8±0.83) values (t(4) = 0.090, p < 0.001).

Similarly, the pre-test (6±0.53) and post-test (3.9±0.7) values of middle-aged adult stoma patients showed a significant reduction (t(10) = 0.001, p < 0.001). The pain intensity of older adult stoma patients also showed a significant reduction from pre-test (7±1) to post-test (3.6±1.15) values (t(2) = 0.000, p < 0.001) (Table 5).

**Table 5:** Pre-test and post-test values determined based on the Numerical Pain Rating Scale

Numerical Pain Rating Scale	Pre-test		Post-test		t value	p value
	Mean	SD	Mean	SD		
Young adults (18-25 years)	7.5	0.7	4.0	0.0	7.0	0.090
Adults (26-44 years)	6.2	0.83	3.8	0.83	0.090	0.001
Middle-aged adults (45-59 years)	6.0	0.53	3.9	0.7	0.001	0.001
Older adults (60 years and above)	7.0	1.0	3.6	1.15	0.000	0.010

The pre-test (44.5±2.12) and post-test (56.0±2.82) values of quality of life (measured using the Stoma-QoL questionnaire) among young adult stoma patients were statistically significant (t(1) = -23.0, p < 0. 001). Likewise, a significant improvement was observed in quality of life among adult stoma patients from pre-test (41.0±3.39) to post-test (54.6±3.78) values (t(4) = 0.028, p < 0.001). Similarly, the quality of life among middle-aged adult stoma patients showed a significant improvement from pre-test (44.8±5.9) to post-test (54.0±5.9) values (t(10) = 0.000, p < 0.001) and also the pre-test (33.3±2.3) and post-test (42±1.73) values of older adult stoma patients showed a significant improvement for quality of life (t(2) = 0.000, p < 0.001) (Table 6).

**Table 6:** Pre-test and post-test values after administering the Stoma-QOL Questionnaire to the participants

Stoma-QoL Questionnaire	Pre-test		Post-test		t value	p value
	Mean	SD	Mean	SD		
Young adults (18-25 years)	44.5	2.12	56.0	2.82	-23.0	0.028
Adults (26-44 years)	41.0	3.39	54.6	3.78	0.028	0.000
Middle aged adults (45-59 years)	44.8	5.9	54.0	5.9	0.000	0.000
Older adults (60 years and above)	33.3	2.30	42.0	1.73	0.000	0.001

This demonstrates that specific mobility exercises received a higher score among stoma patients after four weeks of intervention with a statistically significant difference (p < 0.001) for pain intensity and quality of life. This is considered to show a significant reduction in pain on the Numerical Pain Rating Scale and an improvement in quality of life on the Stoma-QoL Questionnaire among ostomy patients.

## Discussion

The primary aim of the study was to ascertain the effectiveness of specific mobility exercises on patients who underwent stoma surgery. The study was performed for a duration of four weeks. The results were measured using the Numerical Pain Rating Scale and Stoma-QoL questionnaire at the start and end of an intervention. Patients experiencing pain and reduced quality of life were found to experience a beneficial effect by performing the specific mobility exercises following surgery as indicated by the mean scores of the Numerical Pain Rating Scale, which were significantly lower denoting a lowering of their pain, and the mean scores of Stoma-QoL questionnaire, which were significantly higher, indicating an improvement in their quality of life.

Recent studies showed that when compared to patients with stoma who do not participate in the exercise program, patients with stoma who attended the exercise program experienced improvements in their quality of life [19]. According to national guidelines from the Association of Stoma Care Nurses, in order to restore the abdominal wall, patients should be advised on suitable exercises for the abdominal and core muscles following surgery. Additionally, guidelines from the World Cancer Research Fund suggested that cancer patients should adhere to the general population’s recommendations regarding physical activity, diet, alcohol consumption, and smoking [20]. From all these studies, we can understand that exercises are important after surgery, especially for stoma patients to maintain their physical and mental well-being. So, stoma patients have to be advised of specific mobility exercises without any heavy lifting activities and these exercises can also be incorporated into the daily life among ostomy patients. Heavy lifting activities and body building must be avoided at earlier stages post-surgery, as it mainly may cause complications, especially parastomal hernia.

In 2017, a study documented that patients were afraid of getting physically active and performing exercises [12]. However, in the present study, the patients were responsive in continuing the exercise program except for the young adult stoma patients as they were found to be anxious and depressed over a stoma as it has changed their lifestyle, overall self-image, their desire to travel, and to play sports, which was reflected in the NPRS scores, which were not statistically significant for pain (t(1) = 7, p > 0.001). So the patients and caregivers should be encouraged to participate in the post-operative counselling to understand the challenges they may face, which would enhance the intervention uptake. The site of the stoma should be discussed before surgery, which would also reduce their anxiety and depression.

In other relevant studies, it has been shown that for patients who had decreased mobility, their quality of life was affected negatively [21]. Our present study is more advantageous as the intervention of specific mobility exercises activated the muscles and helped mobilize the patients, which in turn reduced their immobilization period as a result of stoma surgery. Ultimately, the exercises also contributed to reducing pain and enhancing the quality of life among ostomy patients.

In a previous study, walking for longer than 60 minutes a week among patients who had inflammatory bowel disease improved their quality of life related to their physical and mental health [16]. 

Patients who underwent stoma surgery after being diagnosed with colorectal cancer reported reduced overall quality of life, decreased self-image, decreased social engagement, increased anxiety and mental illness, and worse quality of life related to health [4]. On the other hand, it was concluded that progressive muscle relaxation training could enhance the psychological well-being and overall quality of life among patients with colorectal cancer when the intervention was given for 10 weeks [22]. When specific mobility exercises were given for four weeks for patients with colorectal cancer and inflammatory bowel disease, these exercises kept them engaged with their daily life, helped avoid negative thoughts, and also prevented them from social withdrawal, which ultimately improved their quality of life. Future studies can be done by combining these interventions, which may have a greater impact among patients with inflammatory bowel disease and colorectal cancer following their surgery.

Ultimately, when patients performed these exercises, it was reported that their pain was reduced and their quality of life improved [23]. Furthermore, it is highly advised for health care professionals to encourage patients to perform exercises after their surgery. They can also educate patients about the importance of exercises and the possible complications that may arise if exercises are not performed. These specific mobility exercises are recommended following stoma surgery as it is considered to enhance recovery for patients. Patients should regain confidence and should be encouraged to perform activities in the following weeks to maintain their overall quality of life after their surgery.

## Conclusions

Our study found evidence that four weeks of specific mobility exercises in line with general medical treatment showed a significant reduction in pain and an improvement in quality of life among stoma patients. However, in earlier stages, patients were likely experiencing pain and discomfort, making it difficult to perform exercises effectively and safely. The majority of the patients in our study were male and future studies need to include a more diverse ostomy patient population. Based on the conditions, our study included only a fewer patients with inflammatory bowel disease, which is also one of the limitations of the study. Future studies can be done by equally distributing gender and patient conditions. We emphasize the importance of randomizing patients into the experimental and control groups and including a combination of other exercises in rehabilitation for patients following stoma surgery.
